# The genus *Indigofera* (Leguminosae) in New Caledonia: two new species and a key for the species

**DOI:** 10.3897/phytokeys.119.32221

**Published:** 2019-03-20

**Authors:** Marc Pignal, Luciano Paganucci de Queiroz

**Affiliations:** 1 Institut de Systématique, Évolution, et Biodiversité (UMR 7205 - CNRS, MNHN, UPMC, EPHE), Muséum national d’Histoire naturelle, Sorbonne Universités, case postale 39, 57 rue Cuvier, 75231 Paris cedex 05, France Sorbonne Universités Paris France; 2 Departamento de Ciências Biológicas, Universidade Estadual de Feira de Santana, Av. Transnordestina s/n, 44031-460, Feira de Santana, BA, Brazil Universidade Estadual de Feira de Santana Feira de Santana Brazil

**Keywords:** Indigofereae, Fabaceae, New-Caledonia, Papilionoideae, taxonomy, Indigofereae, Fabacées, Nouvelle-Calédonie, Papilionoidées, taxonomie

## Abstract

*Indigoferamonieriana* M.Pignal & L.P.Queiroz, **sp. nov.** and *Indigoferadumbeana* M.Pignal & L.P.Queiroz, **sp. nov.**, two new species from New Caledonia, are described and illustrated. Both new species have been collected for a long time, but most herbarium specimens were named as the Australian species *Indigoferaaustralis*, even though they clearly stand apart from this species and the other New Caledonian species of the genus. *Indigoferamonieriana* can be diagnosed by the tall virgate shrubby habit, leaves with an articulate rachis and 7–11 widely obovate to orbiculate leaflets with greyish undersurface and almost invisible venation. *Indigoferadumbeana* can be recognized by the arborescent habit, leaves with 15–19 elliptical leaflets, small, c. 6 mm long flowers, and ellipsoid seeds. Preliminary IUCN assessments are provided for both species. A key is provided for all species of *Indigofera* recorded from New Caledonia.

## Abstract (fr)

*Indigoferamonieriana* M.Pignal & L.P.Queiroz, **sp. nov.** et *Indigoferadumbeana* M.Pignal & L.P.Queiroz, **sp. nov.**, deux nouvelles espèces de Nouvelle-Calédonie, sont décrites et illustrées. Toutes deux sont récoltées depuis longtemps, mais la plupart des spécimens d’herbier sont rapportés à *Indigoferaaustralis*, une espèce australienne, même si elles se distinguent clairement de cette espèce et des autres espèces du genre de la Nouvelle-Calédonie. *Indigoferamonieriana* peut être caractérisée par son port arbustif élevé, ses feuilles avec un rachis articulé et 7–11 folioles largement obovales à orbiculées, une face abaxiale grisâtre et une nervation presque invisible. *Indigoferadumbeana* se reconnaît à son port arborescent, à ses feuilles avec 15–19 folioles elliptiques, à ses fleurs de petite taille (environ 6 mm de long) et à ses graines ellipsoïdes. Des statuts UICN préliminaires sont proposés. Une clé est fournie pour toutes les espèces d’*Indigofera* répertoriées en Nouvelle-Calédonie.

## Introduction

New Caledonia is a sui generis French collectivity located in the southwest Pacific Ocean, 1210 km east of Australia. It is an archipelago of about 18,600 km^2^ in Melanesia subregion with a mostly subtropical climate. Most of New Caledonia’s native vegetation can be classified into three major types: dense rainforests, savannas and maquis vegetation, the latter a kind of low, sclerophyllous, evergreen vegetation largely restricted to ultramafic substrates (Jaffré et al. 2012).

The New Caledonian native flora includes more than 3300 species of seed plants with an extraordinarily rich endemism, including several examples of relictual Gondwanan elements, such as gymnosperms, of which 42 out of 44 native species are endemic (de Laubenfels 1972, Gargominy et al. 2018). The flowering plant families Amborellaceae, Oncothecaceae and Phellinaceae are also restricted to New Caledonia. According to the French taxonomic repository (Gargominy et al. 2018), Leguminosae are represented by 39 genera (5% endemic to the island), 103 species (32% endemic), 6 subspecies and 13 varieties. General endemism in this collectivity is estimated at c. 76.4% (Jaffré 1993) and no doubt many taxa still remain to be described (Morat 2010, Gâteblé et al. 2018). New Caledonia also includes 2008 introduced species (Héquet et al. 2009).

*Indigofera* L. is a legume genus belonging to the tribe Indigofereae, subfamily Papilionoideae (Schrire 2005, LPWG 2017). The genus is characterized by a combination of the presence of medifixed T-shaped hair, pulvinate leaves, axillary simple racemes, anthers with appendiculate connective, and flowers with an explosive pollen display (Hutchinson 1964; de Kort and Thijsse 1984). *Indigofera* is the third largest genus in Leguminosae, embracing c. 750 species with a worldwide distribution, but with a major diversity center in Africa and Madagascar (Schrire 2005; Schrire et al. 2009). In Tropical Asia to Pacific region, the genus is represented by c. 100 species with several species endemic to each region (de Kort and Thijsse 1984; Wilson and Rowe 2004; Schrire 2005).

*Indigofera* was known in New Caledonia by seven species: *I.atropurpurea* Buch.-Ham. ex Hornem., *I.australis* Willd., *I.hirsuta* L., *I.linifolia* (L.f.) Retz., *I.spicata* Forssk., *I.suffruticosa* Mill., and *I.zollingeriana* Miq. Interestingly, all species except *I.australis* are introduced, weedy and widespread plants, contrasting with the high endemicity of the Caledonian flora. *Indigoferaspicata* (as *I.endecaphylla* Jacq.) is used as forage plant (*Sarlin 18*, P03615827). *Indigoferahirsuta*, *I.linifolia* and *I.suffruticosa* are considered invasive by the Pacific Islands Ecosystems at Risk (PIER 2013) and in New Caledonia by Meyer et al. (2006).

The Australian *I.australis* is commonly cited as occurring in New Caledonia (Guillaumin 1936, Jaffré et al. 2002). However, during fieldwork in New Caledonia, one of us (MP) had the chance to survey plants that match specimens identified as *I.australis*. After a careful review of morphological variation of all New Caledonian specimens identified as *I.australis* and their comparison to the Australian ones, we concluded that they belong to the two new species described in this work and that *I.australis* does not occur in New Caledonia.

## Materials and methods

### Plant material

We studied the material kept at the National Herbarium of Paris (**P**) and the IRD herbarium of New Caledonia in Nouméa (**NOU**).

### Measurements, observations and abbreviations

We used the tool “collaboratoire” of the national French infrastructure e-ReColNat (ANR-11-INBS-0004) for specimen comparisons. All measurements were taken on adult structures. We took measurements using a stereomicroscope, based only on fully developed and mature organs from dried specimens except for rehydrated floral parts. Extremes of variation are presented in descriptions. Virtual herbarium can be consulted on the research infrastructure RECOLNAT (https://explore.recolnat.org/search/botanique/type=index), P herbarium (https://science.mnhn.fr/institution/mnhn/collection/p//list?lang=en_US) and NOU herbarium (http://herbier-noumea.plantnet-project.org/list.php).

We used the following abbreviations in examined material to indicate the phenological state of the specimen: bt. (with flower buds); fl. (flowering); fr. (fruiting).

### Geographical tools

The distribution map was generated in ArcGis 9.3 software (ESRI 2008), based on data from specimen labels. For old material where the coordinates do not appear, data points were calculated from the data on the specimen labels with the help of the site https://www.geoportail.gouv.fr/carte. The extent of occurrence (EOO) and area of occupancy (AOO) were assessed using GeoCat (Geospatial Conservation Assessment Tool; Bachman et al. 2011) and the preliminary conservation status was assessed using IUCN (2017) criteria. Status will be submitted to IUCN New Caledonian Red List Authority to validate it and make an official IUCN assessment.

## Results

### Key for the New-Caledonian species of *Indigofera*

**Table:** 

1	Leaves simple, elliptic to linear; racemes contracted, to 15 mm long, the flowers tightly clustered in the leaf axil; calyx lobes longer than the tube	*** I. linifolia ***
–	Leaves imparipinnate with three to many leaflets; racemes elongated; calyx lobes shorter or equalling the tube	**2**
2	Leaflets alternate	*** I. spicata ***
–	Leaflets opposite	**3**
3	Inflorescence much shorter than the subtending leaf; pod curved and descending, deflexed against the raceme axis	*** I. suffruticosa ***
–	Inflorescence equaling or longer than the subtending leaf; pod straight, spreading or ascending	**4**
4	Branches, petiole, leaf rachis, inflorescences and pod covered by erect, long (c. 1 mm long) and dark hairs	*** I. hirsuta ***
–	Branches, petiole, leaf rachis and inflorescences covered with adpressed and white or brown hairs, visible only with the aid of a lens, or glabrescent	**5**
5	Leaflets mostly obovate or orbicular with an emarginate apex, whitish or greyish at the lower surface; petals white, sometime tinged pink; standard petal straight or slightly spreading at the anthesis	**6**
–	Leaflets elliptic or ovate with apex acuminate, obtuse or rounded; petals pink, red or purple; standard petal reflexed at the anthesis	**7**
6	Virgate shrub or subshrub, 0.5–2.5 m high; leaves usually with (5-)7–11 leaflets; leaflets mostly 4–12 mm long, venation almost invisible adaxially; leaf rachis articulated; petiolules light brown on dry specimens, of the same color as the rachis; flowers 8–10 mm long	***I.monieriana* sp. nov.**
–	Small tree or shrub, 3–5 m high; leaves with 15–19 leaflets; leaflets mostly 27–32 mm long, secondary veins 6–7 visible on the both sides; leaf rachis not articulated; petiolules dark brown in dry specimens, presenting a different color as the rachis; flowers c. 6.5 mm long	***I.dumbeana* sp. nov.**
7	Leaflet elliptic, apex rounded and mucronate; flowers 8–10 mm long; bracts much longer than the flower bud, persistent; seeds 6–8, circular in cross section and arranged linearly	*** I. atropurpurea ***
–	Leaflet ovate or elliptic-lanceolate, apex acuminate; flowers 4.5–6.5 mm long; bracts shorter than the flower bud, early caducous; seeds c. 16, lens-shaped, compressed in cross section, arranged like a stack of coins	*** I. zollingeriana ***

### Taxonomic Treatment

#### Indigofera monieriana

Taxon classificationPlantaeFabalesFabaceae

M.Pignal & L.P.Queiroz sp. nov.

urn:lsid:ipni.org:names:77195679-1

**Figure 1.**
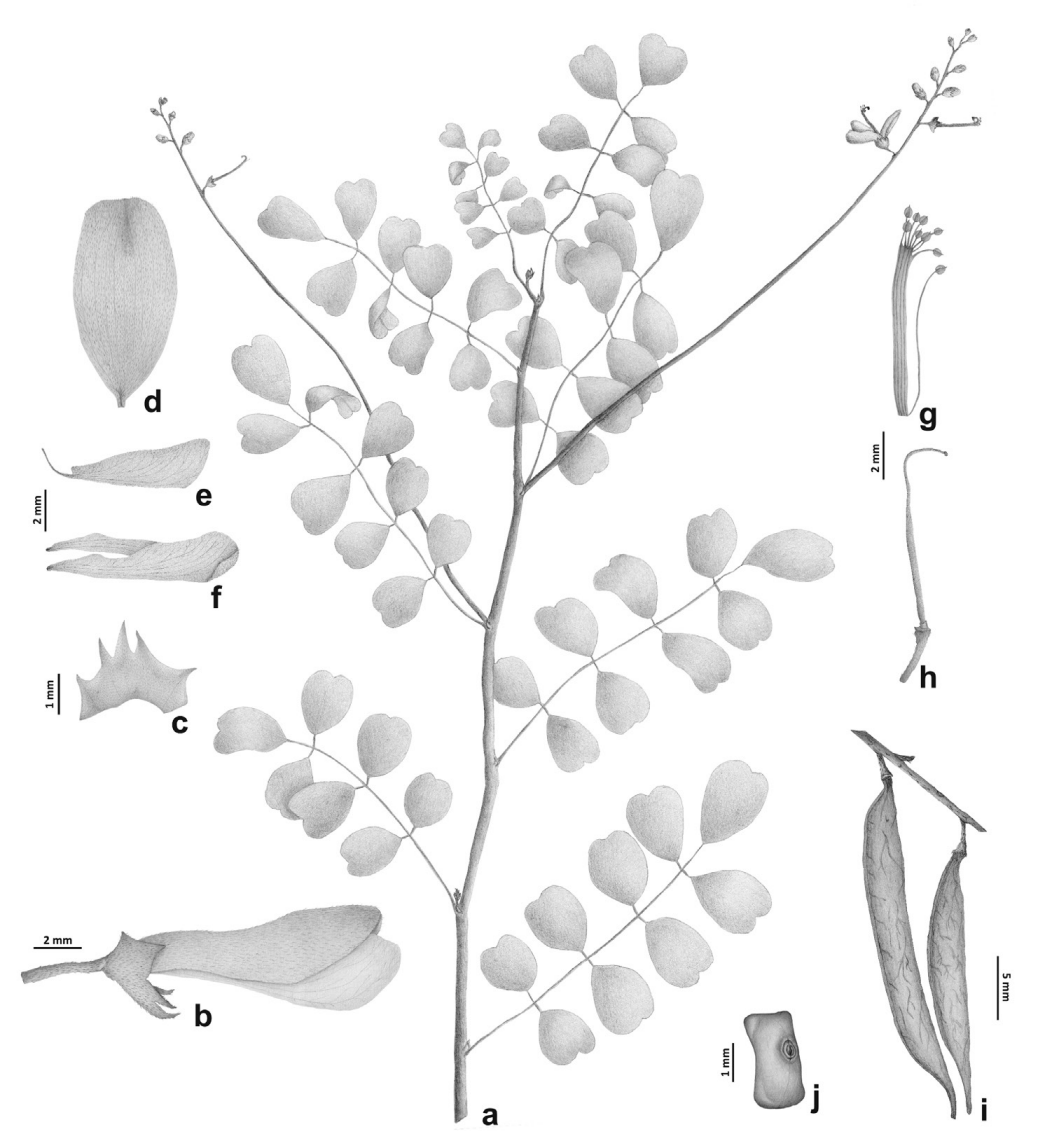
*Indigoferamonieriana* sp. nov. **a** Flowering branch **b** Flower before anthesis **c** Calyx (open) **d** Standard petal **e** Wing petal **f** Keel petals **g** Androecium **h** Gynoecium **i** Fruits **j** Seeds. Drawn by Felipe Santos based on *Pignal 2245*.

Figs 1 , 4a ; Table 1

##### Type.

NEW CALEDONIA. Province Sud, Tontouta, 21°57.9336'S; 166°14.9166'E, pousse dans l’ombre, à la base des arbustes, fleurs blanches, 15 Apr 2004, fl., fr., *M. Pignal 2245* (holotype, P! [P02288351]; isotypes HUEFS! [243158], K!, NOU!).

##### Diagnosis.

*Ab aliis speciecibus* Indigoferarum *Novae-Caledoniae species nova facile differt fruticoso virgato habitu, foliis cum 7–11 foliolis oppositis, late obovalibus vel orbiculatis apice emarginato, nervatione obsolescenti, margine revoluto, pagina infera cinareo-albida, floribus 8–10 mm longis petalis plerumque albis*.

**Table 1.** Comparison between the new species of *Indigofera* from New Caledonia with the Australian *I.australis* and the widespread species *I.zollingeriana*.

	* I. australis *	* I. dumbeana *	* I. monieriana *	* I. zollingeriana *
Habit	Shrub	Shrub or small tree with plagiotropical branches	Virgate shrub	Shrub or small tree
Stipules	Stipules linear	Stipules triangular to falciform	Stipules narrowly triangular or falciform	Stipules linear
Leaf	Leaf 8–10 cm long, rachis only flat (not furrowed) and not articulate, lacking ferruginous colleters fields, at leaflets attachment. Leaflets 17–25.	Leaf, 10.5–11.3 cm, rachis strongly furrowed and not articulate, with brown ferruginous colleters fields, at leaflets attachment. Leaflets 15–19.	Leaf, 3–6 cm long, rachis furrowed and articulate dense colleters fields, dark ferruginous, at leaflets attachment. Leaflets 7(–11).	Leaf 23–26 cm long. Leaflets 11–23.
Leaflet	Petiolule dark brown on dry specimens, same or different color as the rachis; leaflets green below, usually 10–40 mm long, mostly elliptical, apex rounded or obtuse margins thick but not revolute, secondary and sometimes also tertiary venation visible as darker lines at both (but mostly at lower) surfaces, 6–8 pairs of secondary veins.	Petiolule dark brown on dry specimens, different as the rachis; leaflets discolor, greyish green below, usually 17–32 mm long, obovate, apex slightly emarginate, mucronate, margins not revolute, secondary and sometimes also tertiary venation visible at both surfaces, 6–7 pairs of secondary veins.	Petiolule light brown on dry specimens, same color as the rachis; leaflets discolor, whitish or greyish green below, usually 4–12 mm long, mostly obovate or orbiculate with apex emarginate margins slightly revolute discolour, the lower surface grayish venation not visible, 2–4 secondary veins often not visible.	Petiolule dark brown on dry specimens, same color as the rachis; leaflets discolor, greyish green below, usually 35–85 mm long, mostly elliptical-lanceolate with acuminate apex, margins not revolute, c. 10 pairs of secondary veins.
Flower	Calyx truncate; petals pink to purple; standard petal reflexed, keel petals oblong to narrowly obovate.	Calyx 5-lobate, the two upper lobes very short; petals white; standard petal patent, keel petals obovate.	Calyx 5-lobate, the lower lobe as long as or longer than the tube standard petal straight keel petals narrowly obovate.	Calyx 5-lobed, the lower lobe much shorter than the tube; petals pink to red; standard petal straight, keel petals oblong with a 90° upcurved apex.
Pod	Pod straight; endocarp forming translucent envelopes around the seeds; seeds rectangular and arranged linearly.	Pod slightly sinuous; endocarp forming translucent envelopes around the seeds; seeds ellipsoid and arranged linearly.	Pod straight; endocarp forming translucent envelopes around the seeds; seeds rectangular and arranged linearly.	Pod strongly sinuous; seeds naked, lens-shaped and arranged like a pile of coins.

##### Description.

*Virgate shrub* or *subshrub*, 0.5–2.5 m high, with slender and thin stems, young stems flexuous, with short internodes (sometimes up to 3 mm long) at the base, slightly quadrangular becoming terete; indumentum of young branches and leaves of straight, white, adpressed T-shaped hairs. *Stipules* 1–1.5 × 0.4–0.5 mm, narrowly triangular to falciform, pubescent. *Leaves* 3–6 cm long, pinnate, (5–)7–11-foliolate, sometimes 3-foliolate towards the base of the stem; petiole furrowed, 5–12 × c. 0.6 mm; rachis furrowed, quadrangular in cross section, articulated, thick black multicellular hairs (colleters) at the leaflets attachments, segments 3–7 mm long; stipels 0.1–0.5 × c. 0.2 mm, brown, thick, forming the 2 lateral apices of the rachis articles; leaflets opposite, widely obovate to orbicular, dark green above, greyish to whitish pale green below, base rounded to obtuse, apex mostly emarginate, rarely rounded, mucronate, the mucron c. 0.2 mm long, brown, margin entire, slightly revolute, secondary veins 2–5, brochidodromous, invisible adaxially, obscure to invisible abaxially; terminal leaflet 7–17 × 6–12 mm, lateral leaflets (4–)9–12 × 4–6 mm; petiolules 0.6–1.5 mm long, of the same color as the rachis. *Inflorescence* a 5–7 cm long raceme (6–13 cm long in fruiting state); peduncle 10–15 mm long, pubescent; bracts 1.1–8 mm long, triangular, shortly acuminate, pubescent; pedicel 2–3 × 0.2–0.25 mm. *Flower* 8–10 mm long; calyx 2–2.5 mm long, campanulate, asymmetrical, 5-lobed, the vexillary (upper) lobes shorter and deltoid, the carinal (lower) lobe longer and acuminate; petals white, sometimes tinged with pink, never red, standard petal c. 8–9.4 × 4.5–5 mm, elliptical or ovate, apex emarginate, pubescent outside with appressed T-shaped hairs, wing petals c. 7–7.5 × 1.5–2.5 mm, slightly shorter than the keel, narrowly obovate to oblong-linear, apex rounded, keel petals 7–9 × 2–2.5 mm, narrowly obovate, spathulate, apex rounded, valvately connate along the lower margin halfway to the tip; androecium diadelphous (9 stamens fused and the vexillary one free), staminal tube 7.5–9 × c. 2 mm; ovary c. 6-ovulate, c. 5.5 mm long, sessile, glabrous, style c. 3.5 mm long, hook-shaped at apex (hook c. 1.2 mm), stigma capitate. *Pod* 27–38 × c. 3 mm, straight, linear, apex acuminate by 2–4 mm, indehiscent or late dehiscent; valves reddish brown in the living plants, glabrescent or with white appressed T-shaped hairs. *Seeds* 2–4, c. 2.5 × 1.2–1.5 mm, rectangular; testa black.

##### Distribution and habitat.

**Figure 2.**
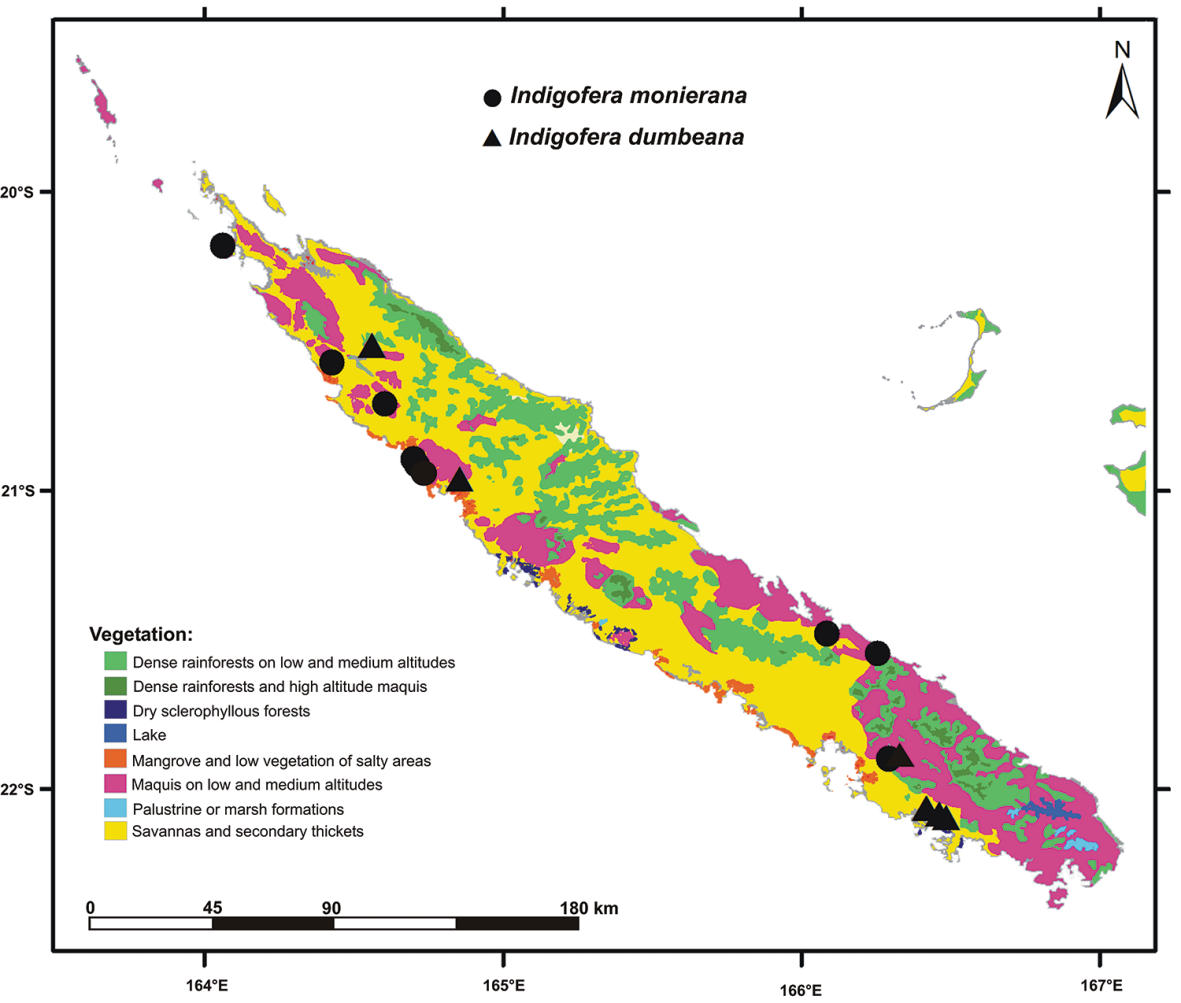
Map of the New Caledonia archipelago showing the major vegetation types (from Jaffré et al. 2012) and the distribution of the new species *Indigoferamonieriana* and *I.dumbeana*.

*Indigoferamonieriana* occurs in shrubby maquis vegetation or low forests on schisto-serpentine soil or ferritic soil. It is found in low altitudes on coastal formations from sea level up to about 700 m. Although it occurs in an open environment, it grows under the shadow of higher bushes (See Fig. 2). Coastal formations, especially sclerophyll forests, have greatly reduced (Bouchet et al. 1995, Jaffré et al. 2002).

##### Phenology.

Flowering from January to March, fruiting from May to November.

##### Etymology.

This species is dedicated to Louis-Guillaume Lemonnier (1717–1799), “*associé de l’Institut, ci-devant membre de l’Académie des sciences, conseiller d’État honoraire, et premier médecin du Roi*” (Cuvier 1861) [Associate of the French Institute, former member of the Academy of Sciences, Honorary State Councilor, and First Physician of the King] who herborised with C. Linnæus, J.-J. Rousseau, Antoine and Bernard de Jussieu. We adopt the spelling “Monier” used by J.-B. Fusée Aublet in his herbarium kept at the Paris Museum and known as “Jean-Jacques Rousseau herbarium” (P-JJR), from the name of its most famous owner.

##### Conservation status.

*Indigoferamonieriana* was provisionally assessed as vulnerable based on IUCN (2017) criterium B as it presents a restricted EOO (7070 km^2^) and AOO (32 km^2^), and its estimated range covers about half of New Caledonia’s main island.

##### Discussion.

Guillaumin (1936) noted that several virgate specimens from New Caledonia could not be reported to the typical *Indigoferaaustralis* and he preferred to determine them as I.australisvar.gracilis DC. However, a careful examination of the type collection of this variety (*Sieber Fl. Novae. Holl. 380*, G, K, P) shows that the New Caledonian material is clearly distinguished from *I.australis* by the less numerous leaflets (5–9 vs. 17–25 in *I.australis*), mostly obovate with an emarginate apex, lower surface whitish or greyish green and inconspicuous venation (vs. leaflets mostly elliptical with acute to rounded apex, green lower surface and raised venation; see Table 1 for further distinctive characters). Besides, *I.australis* is restricted to Australia and, although presenting a broad morphological variation (Wilson and Rowe 2010), its diagnostic features do not overlap with *I.monieriana*. The new taxon is also remarkable for its articulate leaf rachis and its dark ferruginous colleters (multicellular thick secretory hairs) at the insertion of petiolules on the rachis.

##### Paratypes.

**NEW CALEDONIA. Province Nord**: Arbrisseau de 2–3 m de hauteur, NaKéti [=Nakety], sur les collines schisto-serpentineuses, [21°32.814'S; 166°2.532'E], Oct 1869, fr., *B. Balansa 2471* (P [P03615799, P03615800]); Mont Poumé [=Poum], [20°15.282'S, 164°1.584'E], May 1871, fr., *B. Balansa 3332* (P [P03615852, P03615853]; Village de Voh, [20°59.055'S, 164°39.3881'E], 25 Jul 2015, fr., *D. Fleurot 53* (P [P00993550]); Kaala-Gomen, Taom, au-dessus de l’ancienne carrière, Mt. Homédéboa, 720 m, maquis arbustif, sol ferrallitique ferritique sur pente forte érodée, [20°46.998'S, 164°34.002'E], 16 Jan 2007, fl., *R. Barrière & F. Rigault 71* (NOU [NOU016084]); Village de Voh, Tribu de Gatope, presqu’île de Gatope, forêt de 2 à 4 m de hauteur à *Terminalia* et *Homalium*, [20°59.148'S, 164°40.368'E], 17 Nov 2004, fr., *J.-N. Labat et al. 3511* (P [P00454773]; Voh, Vavoutou, forêt sèche, 10 m, [21°0.45'S, 164°41.283'E], 18 Nov 2009, fr., *J.-N. Labat et al. 4050* (P [P00749569]; Voh, Gatope, presqu’île de Gatope, maquis minier. Serpentine. Strate inférieure, [20°59.683'S, 164°40.133'E], 20 Nov 2009, fr., *J.-N. Labat et al.* 4082 (NOU, P [P00749614]; Au-dessus de Gomen, Mt. Kaala, 500–700 m, maquis sur pente raide serpentineuse, [20°38.55'S, 164°23.448'E], 18 Mar 1966, fl., *H.S. MacKee 14586* (NOU [NOU070814], P [P03615845, P03615846, P03615847]; Pente S Mt. Kaala, [20°38.55'S, 164°23.448'E], 27 Mar 1980, fl. *H.S. MacKee 37971* (NOU [NOU070811], P [P03055971]); suffrutex 0,5 met, Montagne de Gomouen, Gatope, [20°58.014'S, 164°39.786'E],1867, fl, fr., *E. Vieillard 2535* (P [P00888525 to P00888532]); **Province Sud**:Collines ferrugineuses situées à l’embouchure du Dotio [=Dothio], [21°36.882'S, 166°12.684'E], 1 Nov 1870, fr, *B. Balansa 3003* (P [P03615854, P03615855], MO); Tontouta: col de Mo, 400 m [c. 21°58'S, 166°11'E], 27 Jan. 1969, fr, *H.S. MacKee 20168* (P [P00888523]); Tontouta [c. 21°58'S, 166°11'E], 25 Dec. 1971, fr, *H.S. MacKee 24758* (P [P03615805]); Basse Tontouta (rive gauche) [c. 21°58'S, 166°11'E], 14 Jan. 1982, fl., *H.S. MacKee 40197* (P [P03615806]) .

#### Indigofera dumbeana

Taxon classificationPlantaeFabalesFabaceae

M.Pignal & L.P.Queiroz sp. nov.

urn:lsid:ipni.org:names:77195680-1

**Figure 3.**
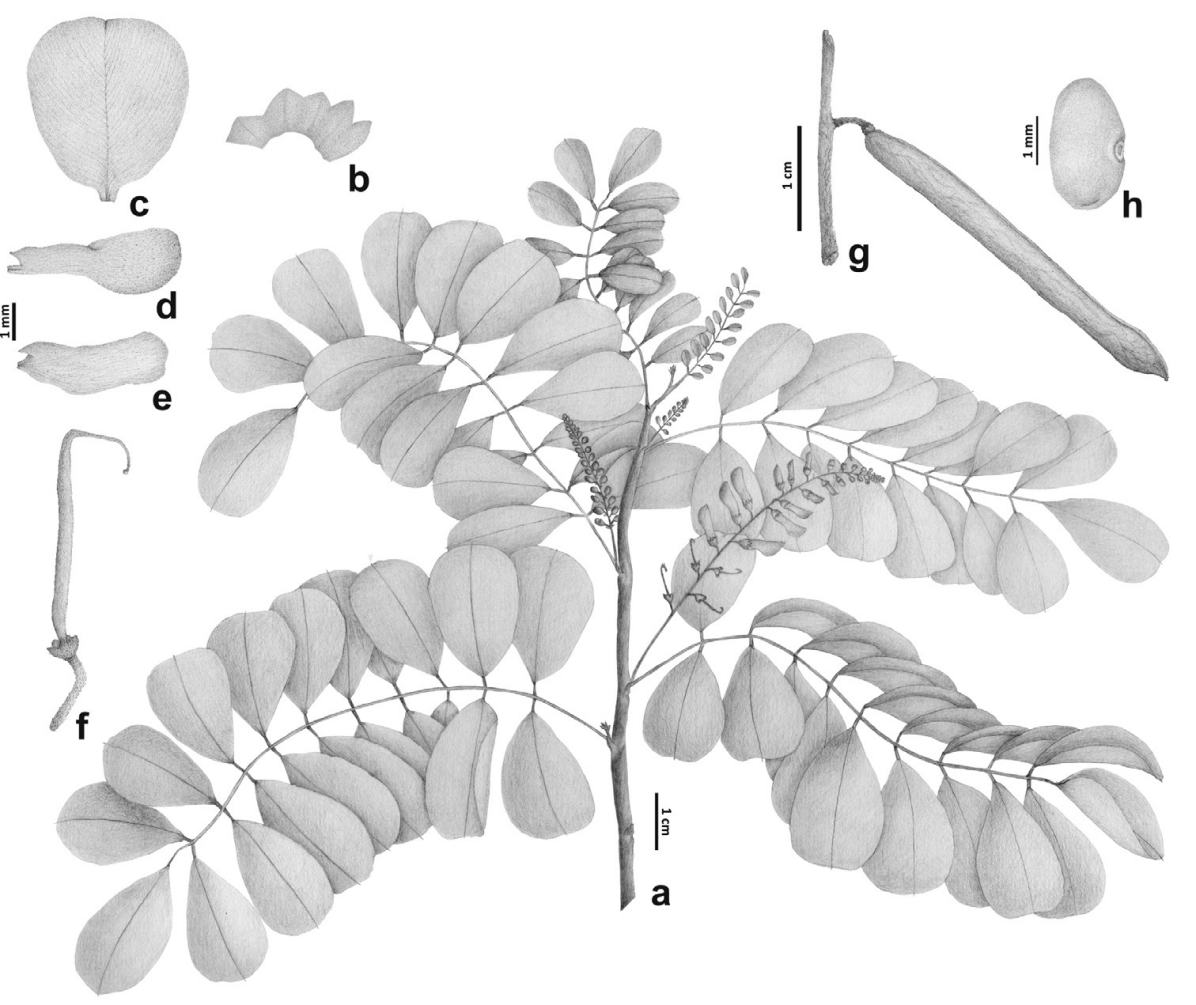
*Indigoferadumbeana* sp. nov. **a** Flowering branch **b** Calyx (open) **c** Standard petal **d** Wing petal **e** Keel petals **f** Gynoecium **g** Fruit **h** Seeds **a–f** after *Balansa 2807***g–h** after *Nothis 440.* Drawn by Felipe Santos.

**Figure 4.**
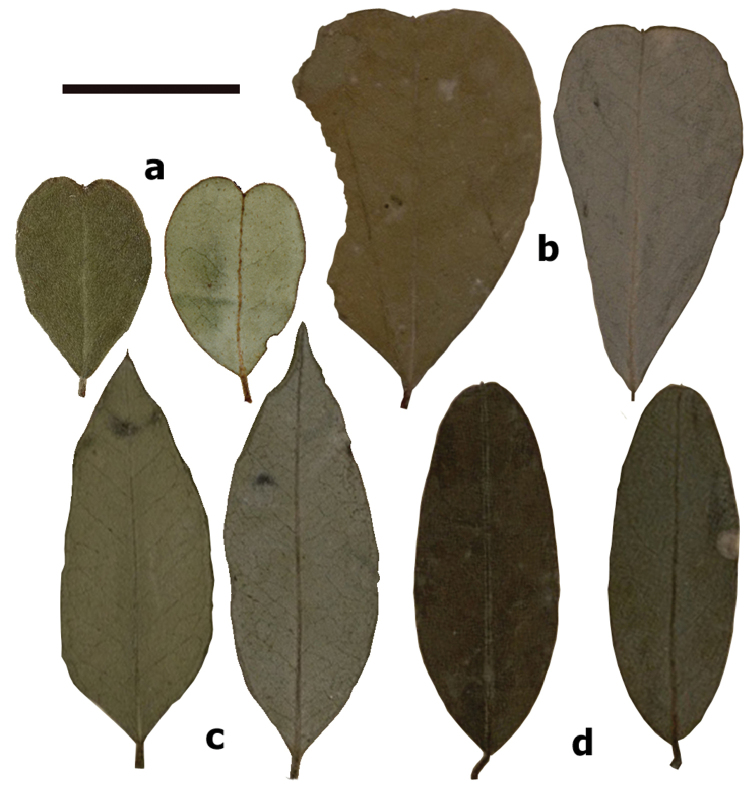
Leaflets comparison of the two new New Caledonian species of *Indigofera* in and their most related species. **a***Indigoferamonieriana* (*M. Pignal 2245*) **b***I.dumbeana* (*B. Balansa 2807*) **c***I.zollingeriana* (*B. Balansa 1222*) **d***I.australis* (*C. Walter s.n.*). Left: adaxial surface, right: abaxial surface. Scale bar: 1 cm.

Figs 2 , 3 , 4b ; Table 1

##### Type.

NEW CALEDONIA. Province Nord, Bois des collines schisteuses près de l’embouchure de la Dumbéa, [22°9.7668'S, 166°26.4336'E], May 1870, bt, fl, *B. Balansa 2807* (holotype: P! [P03615849], isotype, P! [P03615850]).

##### Diagnosis.

Indigoferae zollingerianae *Miq. similis, floribus parvis (c. 4.5‒6.5 mm longis) foliisque cum aliquot foliolis (11‒23), sed brevioribus 10.5–11.3 cm longis foliis cum valde canaliculata rachidi, brevioribus plerumque 17–32 mm longis, ovatis vel obovatis (vs. elliptico-lanceolata) apice obtuso vel leviter emarginato, secundorum nervorum 6–7 paribus, (vs. 9–10 secundorum nervorum paria), fructu tantum 5–6 seminibus contiguis, ellipsoideisque, versus 23–26 cm longa folia leviter vel haud canalicalata rachidi, 35–85 mm longa elliptica vel lanceolata foliola acuminato apice, 9–10 secundorum nervorum paria atque circa 16 nuda in cumulo disposita semina), praecipue differt*.

##### Description.

*Small tree or shrub* 3‒5 m high, branches mostly plagiotropic and horizontal (*Veillon 7138*, P), young stems flexuous, quadrangular or terete, internodes 10‒15 mm long (but c. 5 mm long for the flexuous parts); indumentum of young branches and leaves of straight, white, adpressed T-shaped hairs. *Stipules* 1‒1.5 × c. 0.2 mm, narrowly triangular to falciform, pubescent. *Leaves* 10.5‒11.3 cm long, pinnate, 15‒19-foliolate; petiole 10‒15 mm long, furrowed, sparsely pubescent; rachis yellowish to light brown, strongly furrowed, non articulated and with thin appressed hairs, thick brown multicellular hairs (colleters) at the leaflets attachments, segments at the leaflets attachments 10‒14 mm long; stipels 0.2‒0.3 mm long, setiform, early caducous, mostly absent; leaflets opposite, secondary veins visible on both sides of the lamina, upper and lower surfaces with dense, white hairs; terminal leaflet 27‒34 × 15‒19 mm, ovate to obovate, apex rounded to slightly emarginate, mucronate, base acute, secondary veins 7‒12 pairs, lateral leaflets 20‒17 × 12‒14 mm, obovate, apex slightly emarginate, mucronate, secondary veins 6‒7 pairs, petiolules 1.2‒2 × 0.4‒1.5 mm, dark brown on dry specimens, not furrowed. *Inflorescence* a c. 50 mm long raceme, with more than 40 flowers; peduncle pubescent, quadrangular, c. 10 mm long; pedicel c. 1.5 × 0.2 mm. *Flower* c. 6.5 mm long; calyx c. 1.5 mm long, campanulate, asymmetrical, 5-lobed, the vexillary (upper) lobes shorter and shallow deltoid, the carinal (lower) lobe longer and acuminate; petals white; standard petal c. 5–6 × 4.5 mm wide, obovate, apex slightly emarginate, pubescent outside with apressed T-shaped hairs; wing petals c. 4 × 1.5 mm, slightly shorter than the keel, narrowly obovate to oblong-linear, apex rounded; keel petals 5–5.5 × 2 mm, obovate, apex rounded, valvately connate along the lower margin halfway to the tip; androecium diadelphous (9 stamens fused and the vexillary one free), staminal tube c. 6 × 1.5 mm; ovary c. 5-ovulate, c. 5 mm long, sessile, glabrous, style c. 2.5 mm long, hook-shaped at apex, stigma capitate. *Pod* c. 38 × 3 mm, straight, linear, apex acuminate, indehiscent; valves brown, pubescent with appressed white T-shaped hairs. *Seeds* 5–7, c. 3.5 × 5 mm, ellipsoid; testa black.

##### Distribution and habitat.

*Indigoferadumbeana* grows in lowland forests, mostly in wood edges areas (fide *Veillon 7138*, P and *Veillon 7482*, P).

##### Phenology.

Flowering in March and April, fruiting in May to November.

##### Etymology.

The specific epithet refers to the Dumbéa River in the mouth of which B. Balansa collected the type material.

##### Conservation status.

We assessed *I.dumbeana* as endangered both because it presents small EOO (2358 km^2^) and AOO (20 km^2^), and it is located rather in sclerophyllous forests that are perhaps the most endangered formations in New Caledonia, especially at low elevation (Bouchet et al. 1995). Additionally, this species is known by few and rather old collections which could indicate its rarity in the island.

##### Discussion.

We agree with B. Schrire who annotated in 2004 the P00379654 specimen (*M. Debray 2296)* as a new species allied to *I.australis* Willd. Specimens of *Indigoferadumbeana* were previously referred to *I.australis* by Guillaumin (1936). These species are clearly rendered distinct by the habit as *I.dumbeana* presents plagiotropical, almost horizontal branches, stipules triangular or asymmetrical and falciform (vs. linear), and flowers with a five lobate calyx and white petals (vs. flowers with a truncate calyx and pink to purple petals). *Indigoferadumbeana* is more similar to *I.zollingeriana*, both occurring as a tall shrub or small tree habit with plagiotropical branches, but they are clearly distinct by the fruit straight with rectangular seeds linearly arranged (vs. fruit sinuous with transversely compressed seeds arranged like a stack of coins in *I.zollingeriana*). Additionally, they present important differences in leaf and flower traits as presented in Table 1.

##### Paratypes.

**NEW CALEDONIA.** Tronc grêle, hauteur 4 m, cime légère, croissant en massifs, 200 mètres au dessus du niveau de la mer, localisé, s.d., fr., *I. Pancher s.n.* (P! [P03615856]); **Province Nord**: Forêt derrière Ouéholle, [20°35.316'S, 164°31.464'E], 17 Aug 1967, fr, *A. Nothis 440* (NOU [NOU070806], P! [P02851253]); Pouembout: commun, localisé en lisière de forêt, forêt plate, vers 500 m, forêt dense de moyenne altitude, substrat schistes, [21°1.998'S, 164°49.002'E], 18 June 1992, fr., *J.-M. Veillon 7482* (NOU [NOU070809], P! [P02851258, P02851259]); **Province Sud**: Tontouta [c. 22S; c.166°13'E], 29 Sept. 1975, fr, *M. Debray 2296* (NOU, P [P00379654]; Sur un monticule de la région de Païta, [22°7.95'S, 166°22.566'E], s.d., fr, *I. Pancher (Mus. Néocal. 177)* (P! [P00888524, P03615848, P03615857]); sur les monticules argilo-schisteux de Païta, [22°7.95'S, 166°22.566'E], s.d., fr, *I. Pancher s.n.* (P [P03615844]; Nakutakoin: vallée au sud du pic Jacob, exposition S.W, vers 300 m, en lisière de forêt vallicole (vu aussi à l’intérieur), substrat phtanites, sol brun, [22°0.9'S; 166°25.002'E], 24 Aug 1989, fr., *J.-M. Veillon 7138* (P [P03567480, P03615801], NOU [NOU070810]).

## Supplementary Material

XML Treatment for Indigofera monieriana

XML Treatment for Indigofera dumbeana

## Appendix

**Other examined material**

***Indigoferaaustralis* Willd.: AUSTRALIA.** Nova Hollandia, *s.c. in herb. Delacourt* (P! [P03567468]); New South Wales, Macleay R., *H. Beckler s.n.* (K image seen [K000217365]); Nova Hollandia, *s.c. s.n.*, 1821, (G-DC image seen [G00497647]); Belair, South Australia, [34°59.886'S, 138°37.35'E], fl, Sept. 1902, *M. Koch 568* (P! [P02949887]); Emu Bay & Lea Coast generally, 1864, *J. Milligan 21* (P! [P02949882]); Nouvelle-Hollande, fl, *F.W. Sieber s.n. in herb. van Heurk* (P! [P02949870 p.p.]; Nouvelle-Hollande, 1825, *F.W. Sieber 379* (P! [P02949883, P02949854, P02949868]); Nova Hollandia, 1825, *F.W. Sieber 380* (G image seen [G00418819, G00418820, G00418821], G-DC image seen [G00497743], K image seen [K000217368], P! [P02141690, P02949867]); Mt Macedon (Victoria, Australia), fl, 21 october 1882, *C. Walter s.n.* (P! [P02949870 p.p., P02949889]);

***Indigoferazollingeriana* Miq. VIETNAM.** Tonkin, Ouonbi [Núi Tản Viên, 21°3.6'N; 105°21.6'E], dans les broussailles, fl, 12 Sept. 1885, *B. Balansa 1222* (P! [P02958234]); Lat Son (HN), [20°31.8'N; 105°53.4'E], fl, 19 Aug. 1891, *H. Bon 4859* (P! [P03026363]); Tonkin, Bois de Co-Phah, entre le canal des rapides et Bac-Ninh, fl, 20 Aug. 1891, *B. Balansa 4881* (P! [P03026356]); Tonkin: pr. Lang Son, Cai Kinh, fr., s.d., *A. Chevalier 27682* (P! [P03026361]).
